# Assessment of hypertension management in primary health care settings in Kinshasa, Democratic Republic of Congo

**DOI:** 10.1186/s12913-015-1236-y

**Published:** 2015-12-24

**Authors:** Aimée M. Lulebo, Mala A. Mapatano, Patrick K. Kayembe, Eric M. Mafuta, Paulin B. Mutombo, Yves Coppieters

**Affiliations:** Kinshasa School of Public Health, Kinshasa, Democratic Republic of Congo; School of Public Health, Université Libre de Bruxelles (ULB), Brussels, Belgium; Department of Epidemiology and Bio-statistics, Kinshasa School of Public Health, University of Kinshasa, P.O. Box 11850, Kinshasa, Democratic Republic of Congo

**Keywords:** Hypertension, Management, Primary health care and DRC

## Abstract

**Background:**

Hypertension-related complications have become more diagnosed at secondary and tertiary care levels, in the Democratic Republic of the Congo (DRC), probably indicative of poor management of hypertensive patients at primary health care level. This study aimed to assess the management of hypertension in primary health care settings by using guidelines of the International Forum for Prevention and Control of HTN in Africa (IFHA).

**Methods:**

A multi-center cross-sectional study was carried out in primary health care settings. A total of 102 nurses were surveyed using a structured interview. Mean and proportion comparisons were performed using the *t* Student test and the Chi-square test respectively. The Kinshasa Primary Health Care network facilities were compared with non-Kinshasa Primary Health Care network facilities.

**Results:**

From the 102 nurses surveyed; 52.9 % were female with a mean age of 41.1, (SD = 10) years, merely 9.5 % benefited from in-job training on cardiovascular diseases or their risk factors, and 51.7 % had guidelines on the management of hypertension. Less than a quarter of the nurses knew the cut-off values of hypertension, diabetes and obesity. Merely 14.7 % knew the therapeutic goals for uncomplicated hypertension. Several of the indicators for immediate referral recommended by IFHA were unmentioned. The content of patient education was lacking, avoiding stress being the best advice provided to hypertensive patients. The antihypertensive most used were unlikely to be recommended by the IFHA.

**Conclusions:**

This study showed a considerable gap of knowledge and practices in the management of hypertensive patients at primary health care facilities in Kinshasa pertaining to the IFHA guidelines. We think that task-shifting for management of hypertension is feasible if appropriate guidelines are provided and nurses trained.

**Electronic supplementary material:**

The online version of this article (doi:10.1186/s12913-015-1236-y) contains supplementary material, which is available to authorized users.

## Background

Africa is actually experiencing one of the most rapid epidemiological transitions characterized by a double disease burden with a mix of high incidence of infectious diseases and a growing prevalence of non-communicable diseases NCDs [1, 2]. Moreover 80 % of the NCDs induced deaths occur in the low and middle income countries [3]. Hypertension is among the commoner NCDs. In Sub-Saharan Africa (SSA), the prevalence of hypertension varies from 6 to 48 % [4]. In the Democratic Republic of Congo (DRC), the prevalence of hypertension has increased significantly from 14.2 % in urban areas and 9.9 % in rural areas in 1987 to 26.7 % in 2005 [5, 6]. Hypertension is a major cardiovascular risk factor (CVRF); it increases cardiovascular morbidity and mortality [7].

Health System (HS) related factors have a significant impact on the control of blood pressure control [8, 9]. HS in African countries is essentially oriented to managing infectious diseases. Therefore, health professionals are unprepared to deal with NCDs, given the environment with its limited resources [10, 11]. Thus, cost-effective approaches are needed to lift health systems-level barriers for the management of NCDs in low and middle income countries (LMICs). The task-shifting strategy defined as the rational distribution of primary health care duties from physician to non-physician health care providers is one of these approaches [12, 13]. Previous studies carried out in LMICs have reported the effectiveness of this strategy in the management of HTN [14, 15].

Task-shifting is also considered in the Health policy of the DRC which is based on Primary Health Care (PHC). A Health Center (HC) is run by a nurse. This represents the first level of care, the first-contact setting between patients and healthcare systems. Screening and management of NCDs such as hypertension and diabetes are included in the essential health activities package of HCs [16]. Patient management at the HC should comply with the Ministry of Health’s guidelines. These guidelines, however, do not provide a clear indication on the management of HTN [17]. HCs have to refer patients to the General Referral Hospital (GRH) [16].

Actually, the referral health care facilities register more and more patients with HTN-related complications including stroke and chronic kidney disease (CKD) [18–21]. The Vitaraa study conducted in Southern Kivu (DRC), found that 86.4 % of hypertensive patients were uncontrolled [22]. This shows the poor control of HTN in the country.

In 2003, the International Forum for Prevention and Control of HTN in Africa (IFHA) developed the guidelines for the management of hypertension in SSA countries [23]. These guidelines were adapted from the 2002 WHO Cardiovascular Risk Management Package in low-medium Resource Settings. The IFHA guidelines use the global approach in the management of HTN, which aims to reduce the absolute risk by the management of multiple individual risk factors [24]. These recommendations emphasize the treatment of HTN, with particular attention to cost-effectiveness and affordability in SSA countries [23].

This study aimed to assess the management of Hypertension adhering to the IFHA guidelines in the primary health care facilities.

## Methods

### Study design and population

A multi-center, cross-sectional study was carried in Kinshasa, the capital city of the Democratic Republic of Congo (DRC) in July 2013. A total of 36 HCs were selected using a multiple stage sampling and a total of 102 nurses were surveyed. During the first stage, 12 Health Zones (HZ) were selected out of the 35 HZ situated in the city using simple random sampling technique. During the second stage, in each selected HZ, a list of HCs was generated and HCs were stratified according to their ownership (public, private and confessional). From each category, three HCs were randomly selected. From each selected HC, three nurses involved in curative activities and present on the day of the survey, were interviewed.

The study protocol was approved by the Kinshasa School of Public Health Ethical Committee (ESP/CE/002/2012). All participants gave their verbal informed consent according to the Helsinki Declaration II. Participants’ confidentiality was assured by not recording their identity or the name of health care facilities.

### Variables

Data were collected during face-to-face interviews using a structured questionnaire. Variables were mainly drawn from the IFHA guidelines which have been adapted for the first level of care; with particular emphasis placed on clinical evaluation of risk factors adapted from the 2002 WHO Cardiovascular Risk Management Package in low-medium Resource Settings scenario [25]. The IFHA guidelines have been summarized in terms of main skills expected from care-providers, as shown in Table 1.

**Table 1:** Summary of IFHA recommendations and variables

Skills expected	Recommendations	Variables resulted
Identify cardiovascular risk factors	These cardiovascular risk factors must be explored: patient personal history (CVD and diabetes) and family history of premature CVD, age, sex, smoking, alcohol consumption, physical inactivity, obesity, diet poor in fruits and vegetables, salt intake.	-Knowledge of cardiovascular diseases -Knowledge of cardiovascular risk factors -Knowledge of cut-off values of cardiovascular risk factors (HTN, diabetes and obesity)
Assess cardiovascular risk level.	Patients are stratified in four levels of cardiovascular risk: low, medium, high and very high risk. Patients with high and very high cardiovascular risk must be referred in the next level of care.	Indication for immediate referrals
Perform patient education	Counseling on diet including excessive alcohol intake, physical activity and cessation of tobacco use.	Content of patients’ education
Provide medical treatment	Hydrochlorothiazide in low dosage.	Antihypertensive the most used

### Statistical analysis

Statistical analyses were performed using the statistical package for social sciences (SPSS) version 21.0. Categorical data were summarized as percentages and continuous data as mean and standard deviation (SD). Means and proportions comparisons were performed using respectively Student t-test, Chi-square test and the Fisher-exact test when Chi-square could not be applied. The Kinshasa Primary Health Care network facilities (KPHC) and the non- KPHC network facilities were compared. The KPHC network facilities comprise a constellation of confessional health centers organized around referral hospitals. The KPHC network facilities have worked using an integrated approach in order to improve diabetes and hypertension care as previously described [26]. The non-KPHC network facilities are the other health-care facilities which do not belong to this network.

## Results

### Facilities identification and sample characteristics

We interviewed 102 of 108 nurses (response rate: 94.4 %). As summarized in Table 2, the majority of surveyed health facilities did not belong to the KPHC network (78.0 %). More than half of the nurses were female (52.9 %). Nurses from the KPHC network were older (44.9 ± 9.3 years vs. 40.0 ± 10.0 years) (*p* = 0.043) and had a lower level of qualification (72.7 % vs. 36.2 %) (*p* = 0.002) than those working in the non-KPHC network. In terms of responsibility in the facility, 71.6 % were not the head nurses.

**Table 2:** Sample characteristics and job training

Variables	Overall n (%)	KPHC Network facilities n (%)	Non KPHC Network facilities n (%)	*p*
	*n* = 102	*n* = 22	*n* = 80	
Sex of respondents				
Male	48 (47.1)	8 (36.4)	40 (50.0)	0.256
Female	54 (52.9)	14 (63.6)	40 (50.0)	
Age	Mean ± SD	Mean ± SD	Mean ± SD	
	41.1 ± 10.0	44.9 ± 9.3	40.0 ± 10. 0	0.043^*^
Function	n (102)	(*n* = 22)	(*n* = 80)	0.892
Head nurse	29 (28.4)	6 (27.3)	23 (28.8)	
Others nurses	73 (71.6)	16 (72.7)	57 (71.2)	
Qualification				
High level	57 (55.9)	6 (27.3)	51 (63.8)	0,002^*^
Low level	45 (44.1)	16 (72.7)	29 (36.2)	
Job training				
Yes	84 (82.4)	19 (86.4)	65 (81.3)	0,757
No	18 (17.6)	3 (13.6)	15 (18.7)	
Period of last job training				
1 year or less	61 (72.6)	14 (73.7)	47 (72.3)	0,906
More than 1 year	23 (27.4)	5 (26.3)	18 (27.7)	
Job training on CVD or CVRF	(*n* = 84)			
Yes	8 (9.5)	6 (31.6)	2 (3.1)	0.001^*^
No	76 (90.5)	13 (68.4)	63 (96.9)	
Existence of guidelines	(*n* = 102)			
Yes	58 (56.9)	18 (81.8)	40 (50.0)	0.008 ^*^
No	44 (43.1)	4 (18.2)	40 (50.0)	
Existence of guidelines for management of hypertension	(*n* = 58)			
Yes	30 (51.7)	12 (66.7)	18 (45.0)	0.127
No	28 (48.3)	6 (33.3)	22 (55. 5)	

* means statistically significant

### Job training and existence/availability of guidelines

Table 2 also summarizes the existence of job training and guidelines. Although, 84 nurses declared they had already received in-job training, only a negligible proportion of them (9.5 %) have received in-job training on cardiovascular diseases or on their risk factors (hypertension, diabetes…). Furthermore, the proportion of those who had received in-job training on cardiovascular diseases and on related risk factors was significantly lower in the non-KPHC network facilities compared to the KPHC network facilities (3.1 % vs. 31.6 %) (*p* = 0.001). More of KPHC network facilities were in possession of guidelines for the management of patients than the non-KPHC network facilities (81.8 % vs. 50.0 %) (*p* = 0.008). However, no statistical difference was observed regarding the presence/availability of guidelines for the management of hypertension between both groups (66.7 % vs. 45.0 %) (*p* = 0.127).

### Assessment of health-care providers’ knowledge

Table 3 presents an assessment of health-care providers’ knowledge. More than 50 % of health providers in both groups were able to mention at least one CVD. Nurses from the KPHC network were more capable than those from non KPHC network in naming at least three cardiovascular risk factors (68.2 % vs. 30.0 %) (*p* = 0.001). The most common cardiovascular risk factor mentioned was stress (60.0 %). The cut-off values of BP for HTN (≥140/90 mmHg) was reported by less than half of them and this proportion was lower among nurses of non-KPHC network compared to those of KPHC network (17.5 % vs. 40.9 %) (*p* = 0.040). The cut-off value of diabetes (≥126 mg/dl) was mentioned by less than one fifth of nurses, but this proportion was lower among nurses from non KPHC network (1.3 % vs. 13.6 %) (*p* = 0.031). The cut-off values of obesity’s index (body mass index (BMI) and waist circumference) were almost unknown by nurses in both groups. A very low proportion of nurses (14.7 %) knew that Systolic Blood Pressure (SBP) <140 mmHg and Diastolic Blood Pressure (DBP) <90 mmHg are the therapeutic goals for uncomplicated hypertension. A similar result was found for the therapeutic goals for complicated hypertension (SBP <130 mmHg and DBP <80 mmHg).

**Table 3:** Assessment of health care providers’ knowledge

Variables	Overall n (%)	KPHC Network facilities n (%)	Non KPHC Network n (%)	*p*
	*n* = 102	*n* = 22	*n* = 80	
CVD knowledge				
Have cited at least one	59 (57.8)	14 (63.6)	45 (56.3)	0.534
Did not cite any	43 (42.2)	8 (36.4)	35 (43.7)	
CVRF knowledge				
Have cited 3 or more	39 (38.2)	15 (68.2)	24(30.0)	0.001^*^
Have cited less than 3	63 (61.8)	7 (31.8)	56 (70.0)	
CVRF mentioned				
Hypertension	33 (32.4)	8 (36.4)	25 (31.3)	0.650
Diabetes	16 (15.7)	8 (36.4)	8 (10.0)	0.006^*^
Obesity	25 (24.5)	9 (40.9)	16 (20.0)	0.043^*^
Tobacco use	20 (19.6)	4 (18.2)	16 (20.0)	1.000
Alcohol consumption	23 (22.5)	6 (27.3)	17 (21.3)	0.571
Diet rich in fat	16 (15.7)	6 (27.3)	10 (12.5)	0.106
Ageing	10 (9.8)	4 (18.2)	6 (7.5)	0.216
Stress	53 (60.0)	8 (36.4)	45 (56.3)	0.098
Salt intake	19 (18.6)	5 (22.7)	14 (17.5)	0.550
Cut-off values of Hypertension				
Know	23 (22.5)	9 (40.9)	14 (17.5)	0.040^*^
Do not know	79 (77.5)	13 (59.1)	66 (82.5)	
Cut-off value of diabetes				
Know	4 (3.9)	3 (13.6)	1 (1.3)	0.031^*^
Do not know	98 (96.1)	19 (86.4)	79 (98.7)	
Cut-off values of BMI for obesity				
Know	3 (2.9)	1 (4.5)	2 (2.5)	0.521
Do not know	99 (97.1)	21 (95.5)	78 (97.5)	
Therapeutic goals for uncomplicated hypertension				
Know	15 (14.7)	2 (9.1)	13 (16.3)	0.514
Do not know	87 (85.3)	20 (90.9)	67 (83.7)	
Therapeutic goals for complicated hypertension				
Know	3 (2.9)	0 (0.0)	3 (3.8)	1.000
Do not know	99 (97.1)	22 (100.0)	77 (96.2)	

* means statistically significant

### Indication for immediate referral, contents of hypertensive patient education and the most used antihypertensive medication

Table 4 shows that 83.3 % of nurses mentioned at least one indicator for immediate referral according to the IFHA recommendations. Hypertension grade 3, patients with signs of stroke and patients with coma were the most mentioned indications. Table 4 reported also on health providers’ practices concerning hypertensive patient education and antihypertensive medication. Avoiding stress (66.7 %) and salt (53.9 %) were the most mentioned advices/recommendations provided by nurses during hypertensive patient education. The most prescribed antihypertensive drug was Alpha methyldopa, and diuretics, namely Furosemide and hydrochlorothiazide.

**Table 4:** Indication for immediate referral, contents of hypertensive patient education and the most used antihypertensive medication

Variables	Overall n (%)	KPHC Network facilities n (%)	Non KPHC Network n (%)	*P*
Indications for immediate referral according to the IFHA recommendations				
Mentioned at least one indication	85 (83.3)	19 (86.4)	66 (82.5)	1.000
Mentioned none	17 (16.7)	3 (13.6)	14 (17.5)	
Patient education content				
Avoid stress	68 (66.7)	11 (50.0)	57 (71.3)	0.061
Diet without salt / Low-salt diet	55 (53.9)	13 (59.1)	42 (52.5)	0.583
Drink of plenty water	13 (12.7)	3 (13.6)	10 (12.5)	1.00
Take a rest	25 (24.5)	3 (13.6)	22 (27.5)	0.181
Avoid coffee	11 (10.8)	3 (13.6)	8 (10.0)	0.699
Avoid alcohol	16 (15.7)	2 (9.1)	14 (17.5)	0.512
Respect taking medications and follow-up visits	23 (22.5)	9 (40.9)	14 (17.5)	0.040^*^
Avoid foods high in saturated fat	13 (12.7)	1 (4.5)	12 (15.0)	0.289
Physical activity	19 (18.6)	7 (31.8)	12 (15.0)	0.118
The most antihypertensive used				
Aldomet	41 (40.2)	3 (13.6)	38 (47.5)	0.000^*^
Furosemide	24 (23.5)	1 (4.5)	23 (28.8)	
Esidrex	16 (15.7)	15 (68.2)	1 (1.3)	
Sub lingual adalat	15 (14.7)	1 (4.5)	14 (17.5)	
Others	7 (6.9)	2 (9.1)	4 (5.0)	

## Discussion

This study shows a considerable gap in knowledge and practices among nurses in their management of hypertension according to the IFHA guidelines. Secondly, few health-care providers declared having used the guidelines for the management of HTN. Although, KPHC providers were more knowledgeable than those from non-KPHC facilities, their knowledge and practices were, however, sub-optimal according to the IFHA guidelines.

The lack of an adequately trained health workforce for the management of non-communicable diseases is described as a challenge for SSA countries [27, 28]. This is corroborated by our findings where it was reported that a low proportion of nurses have received in-job training on cardiovascular diseases or on their risk related factors while a majority of them have reported to have already received in-job training probably on infectious diseases.

In the global approach, the knowledge of cardiovascular risk factors is essential for patients’ cardiovascular risk assessment but also for the patients’ management which must be global. In this study, we found that a low proportion of nurses were knowledgeable on cardiovascular diseases and their risk related factors. Stress was the most cardiovascular risk related factor mentioned and its prevention the commonest advice provided to hypertensive patients; whereas the commoner cardiovascular risk factors such as tobacco consumption, unhealthy diet including excessive alcohol intake and physical inactivity were less mentioned. It is described that the control of these CVRFs has a favourable effect on blood pressure [29]. Counseling by health providers can have a significant impact in motivating and supporting patients’ behavioural change; unfortunately very often health providers inappropriately counsel patients about their life style as also reported by previous studies [30, 31].

This study indicates that the index of obesity was unknown by almost all care-providers. Nevertheless, it is established that obesity is a predictor of mortality among hypertensive patients and that weight loss significantly decreases blood pressure [20, 32]. It suggests that health providers do not place emphasis on evaluating clearly patients’ nutritional status. This finding is corroborated by Mongati et al. who reported that none of the health workers interviewed in their study knew how to measure waist circumference [31]. The awareness of therapeutic goals is a major element for preventing clinical inertia among healthcare providers and their ignorance is a factor in the poor control of blood pressure. Clinical inertia is defined as the failure of health-care providers to initiate or to intensify therapy when therapeutic goals are not reached [33]. Mendis et al. found that a lower proportion of non physician health care workers (NPHWs) knew the goals of treatment for uncomplicated hypertension compared to physicians (39 % vs. 75 %) [34]. This study provides an even worse conclusion than Mendis et al.

According to the IFHA guidelines, patients with a high or very high cardiovascular risk should be referred to a higher health care level. Among patients with a high cardiovascular risk are included hypertensive patients with antecedents of diabetes, cardiovascular diseases, CKD; patients with hypertension grade 1 or 2 associated to 3 or more other cardiovascular risk factors and all patients with hypertension grade 3 [18]. Some of them, namely patients with HTN grades 3, patients with signs of stroke and patients in coma have been the most mentioned indications for immediate referral among the surveyed nurses. Subsequently, the management of this category of patients should not be realized at primary health level but at a General Referral Hospital or at the highest level of health care. This study shows that some patients with a high or very high cardiovascular risk are being managed at the HC level which provides only an essential package of care, insufficient for managing patients in this category. This could explain the frequency of hypertension-related complications registered at the high care level.

Furthermore, this study showed that Alpha methyldopa, Furosemide and Thiazides were the most administered antihypertensive drugs by nurses; this finding corresponds almost to that of Mendis et al. [34], whereas the IFHA guidelines indicate that the anti-hypertensive drugs with central action like Methyldopa should only be used for resistant hypertension [23]. Also, it is more expensive than hydrochlorothiazide [34]. On the other hand, Furosemide is also recommended according to the IFHA guidelines for selected indications including congestive heart failure, advanced renal disease or refractory hypertension [23] and not for all patients.

Despite the fact that nurses of KPHC network had lower qualifications than others, their knowledge and practices were better probably because these healthcare providers received in-job training and were in possession of guidelines with a global approach that recommends hydrochlorothiazide as the first line drug. The non-KPHC network facilities had their guidelines included in the guidelines of care at Health Centers produced by the DRC Ministry of Health. These guidelines, place the diagnosis of hypertension in the symptoms’ context notably headache or coma and recommends Furosemide associated with diazepam as antihypertensive medication. The DRC Ministry of Health recommends to HCs to manage hypertension but it does not provide them with suitable resources. We think that the task-shifting for hypertension management is feasible in DRC if appropriate guidelines are produced and nurses trained like reported by previous studies (14, 15).

However, this study has some limitations. The first limitation is related to the setting and small sample size which makes findings unrepresentative of the country. There is also a limitation from data collection; nurses surveyed self-reported their practices which were not observed. To the best of our knowledge, this study is the first to have assessed the management of HTN at the primary level of care facilities including also facilities which do not belong to the KPHC Network. In addition, this study can be a starting point for improving the management of HTN in the DRC.

## Conclusion

This study showed a considerable gap in knowledge and practices related to management of hypertensive patients at primary health care facilities in Kinshasa according to the IFHA guidelines. This situation was mainly due to the lack of in-job training and the non-optimal quality of therapeutic guidelines on the management of hypertension. These findings imply that the improvement of the healthcare service delivery for hypertension requires that healthcare providers be trained, and that appropriate therapeutic guidelines are designed.

### Availability of supporting data

The data set supporting the results of this study is available in an additional file.

## Additional file

Additional file 1:
**Dataset.** This file contains data supporting the results of this study (http://dx.doi.org/10.5061/dryad.r7082).

## Abbreviations

CKD: Chronic kidney disease
CVD: Cardiovascular diseases
CVRF: Cardiovascular risk factor
DBP: Diastolic blood pressure
DRC: Democratic Republic of Congo
GRH: General Referral Hospital
HC: Health Center
HS: Health system
HTN: Hypertension
HZ: Health zones
IFHA: International forum for prevention and control of hypertension in Africa
KPHC: Kinshasa Primary Health Care
LMICs: Low and middle income countries
NCDs: Non-communicable diseases
NPHWs: Non physician health care workers
PHC: Primary Health Care
SBP: Systolic blood pressure
SD: Standard deviation
SSA: Sub-Saharan Africa
WHO: World Health Organization
